# Bone Dielectric Property Variation as a Function of Mineralization at Microwave Frequencies

**DOI:** 10.1155/2012/649612

**Published:** 2012-04-19

**Authors:** Paul M. Meaney, Tian Zhou, Douglas Goodwin, Amir Golnabi, Elia A. Attardo, Keith D. Paulsen

**Affiliations:** ^1^Thayer School of Engineering, Dartmouth College, Hanover, NH 03755, USA; ^2^Kuang-Chi Institute of Advanced Technology, Shenzhen, Guangdong 518000, China; ^3^Dartmouth-Hitchcock Medical Center, Lebanon NH, 03756, USA

## Abstract

A critical need exists for new imaging tools to more accurately characterize bone quality beyond the conventional modalities of dual energy X-ray absorptiometry (DXA), ultrasound speed of sound, and broadband attenuation measurements. In this paper we investigate the microwave dielectric properties of ex vivo trabecular bone with respect to bulk density measures. We exploit a variation in our tomographic imaging system in conjunction with a new soft prior regularization scheme that allows us to accurately recover the dielectric properties of small, regularly shaped and previously spatially defined volumes. We studied six excised porcine bone samples from which we extracted cylindrically shaped trabecular specimens from the femoral heads and carefully demarrowed each preparation. The samples were subsequently treated in an acid bath to incrementally remove volumes of hydroxyapatite, and we tested them with both the microwave measurement system and a micro-CT scanner. The measurements were performed at five density levels for each sample. The results show a strong correlation between both the permittivity and conductivity and bone volume fraction and suggest that microwave imaging may be a good candidate for evaluating overall bone health.

## 1. Introduction

Osteoporosis is a major health problem for roughly 55% of the US population of 50 years of age or older. It is characterized by low bone mass and structural deterioration which leads to increased fragility and risk of fracture. Fifty percent (50%) of women and 25% of men over age 50 will have an osteoporosis-related bone fracture. The most typical fractures occur in the hip, spine, wrist, and ribs, of which the hip and vertebral fractures can require long-term care and even cause death in as many as 24% of hip fracture cases [1]. This dynamic aspect of bone physiology may facilitate the use of dielectric interrogation as a means of imaging bone health.

Assessing bone health may be a particularly good opportunity to exploit dielectric properties for screening, diagnosis, and in the overall management of bone treatment. In parallel with previous broadband tissue dielectric property studies [2–11], bone has received special attention. For instance, clinicians have used electrical currents to stimulate bone growth for several decades [12–15]. With such treatment it is essential to both understand the reaction of the bone to electrical stimulation as well as understand the tissue dielectric properties for guiding the therapy. In addition, the dielectric properties, themselves, may provide clinically useful information with respect to assessing overall bone health as in the case of osteoporosis and monitoring osteogenic response to treatment. These properties have been studied extensively up to 5 MHz [16–19]; however, tests beyond this frequency have not proved useful to date [20]. At lower frequencies, electrical impedance spectroscopy (EIS) techniques, including parallel plate capacitance cells, have been used to retrieve accurate dielectric properties [21, 22]. In fact, measurements have been made using conventional open-ended coaxial dielectric probes at frequencies as high as 3 GHz, but remain unpublished. The primary reason is that the dielectric probe technique is inherently ill-suited to this type of measurement. While researchers have performed experiments to determine the proximal limits of heterogeneities when testing homogeneous materials and liquids using these probes [23], first-hand experience suggests that measurements with these probes on inhomogeneous targets are dominated by the tissue in direct contact with the probe. Given the heterogeneous nature of trabecular bone samples and the potential for property and texture variations between the less disturbed internal zones and the bone surfaces because of the extensive manipulation involved in preparing the samples, it is not surprising that these probe measurements have been inconclusive. More recent *in vivo *animal studies by Gabriel et al. [8] indicate that dramatic dielectric property changes occur with age in bone (not seen in other tissue types). This dynamic aspect of bone physiology may facilitate exploitation of dielectric interrogation as a means of bone health imaging.

The baseline studies by Gabriel et al. [5–7] and others have proved useful in establishing nominal, frequency-dependent property ranges for different tissue types and have set the stage for further investigation of whether variations in individual tissue dielectric properties can be predictive of various maladies. Studies by Joines et al. [24] and Lazebnik et al. [25, 26] have explored whether tumors exhibit dielectric properties distinct from their normal organ. Additionally, at frequencies below 2 GHz where the ionic flow dominates the overall conductivity effect [27, 28], tissue conductivity has been shown to vary linearly with temperature, and this mechanism has been utilized in investigations of noninvasive temperature monitoring in conjunction with thermal therapy [29, 30]. Similar work has been performed in other fields to look at tissue property variations based on physiological phenomena other than cancer. For instance, in comparable studies of ultrasound computed tomography, Sehgal et al. [31, 32] showed that the speed of sound and broadband attenuation varied considerably for liver samples depending on overall fat and tumor content. Given that the only substantive variations in the tissues tested in that study were the fat and water content levels, dielectric mixture laws such as the Maxwell-Fricke relationships would also naturally predict similar variations [3].

In the area of ultrasound, researchers have performed numerous studies of the effect of bone density on the speed of sound and broadband attenuation, and several devices have been developed and approved by the FDA for testing bone health. For instance, the Lunar Achilles system produced by GE Healthcare (Waukesha, WI, USA) and the Sahara Clinical Bone Sonometer manufactured by Hologic (Bedford, MA, USA) are both FDA approved and used routinely in clinical situations. In the ultrasound experiments performed by Wu et al. [33], trabecular bone samples were tested with both dual X-ray absorptiometry (DXA) and ultrasound transmission techniques to assess whether correlations exist between bone mineral density (BMD) and the ultrasound metrics—speed of sound and broadband attenuation. The samples were tested at a baseline with both techniques and at several subsequent times after demineralization through submersion in acidic solvents. The ultrasound measurements were performed with the bone specimens placed in water to assess overall bulk property changes as the hydroxyapatite was progressively removed and artificially replaced by the water. (Note that hydroxyapatite is the main constituent of the mineralized portion of the bone and is generally referred to as bone mineral.)

We have followed a similar path for this dielectric property study except the DXA X-ray tests have been replaced with more exact X-ray micro-CT measurements to determine the bone sample constituent proportions. Furthermore, we have replaced the ultrasound transmission measurements with microwave tomographic images. These tissue samples do not readily lend themselves to standard dielectric probe measurements because the trabecular bone samples are fragile and their surfaces are uneven given their intricate architecture. Instead, we have applied a newly developed microwave tomographic approach utilizing a soft prior regularization that is well suited for testing the bulk properties of small samples of known geometries [34, 35]. The samples were placed in a test tube filled with saline of known size in a predetermined location within the illumination zone of our microwave tomographic system. Prior knowledge of the sample shape, size, and location was applied in the regularization scheme as part of the standard image reconstruction process [34] to recover estimates of the specimen dielectric properties. This measurement approach is not unlike the ultrasound CT technique described by Schreiman et al. [36] for isolated tissue samples where the configuration geometry was also well known. While replacement of the bone marrow with saline does not perfectly mimic the process that normal bone undergoes during aging and/or unnatural bone loss, it does validate the overall notion that tissue bulk dielectric properties are functions of the individual properties and volume fractions of its constituents. *In situ* dielectric changes will likely occur in more complicated patterns, but these measurements may still provide important information on tissue pathology and health.

In this paper we describe the process used in these experiments including the tissue preparation, X-ray CT, and microwave imaging approaches. We illustrate the technique of applying the microwave tomographic method with soft prior regularization to recover accurate values of the dielectric properties of the bone tissue samples and the associated tools used to assess the radiographic bone properties from the micro-CT data. Finally, we present results demonstrating the correlation between the bone dielectric properties and the X-ray data metrics.

## 2. Methods

### 2.1. Bone Sample Preparation

**Figure 1:**
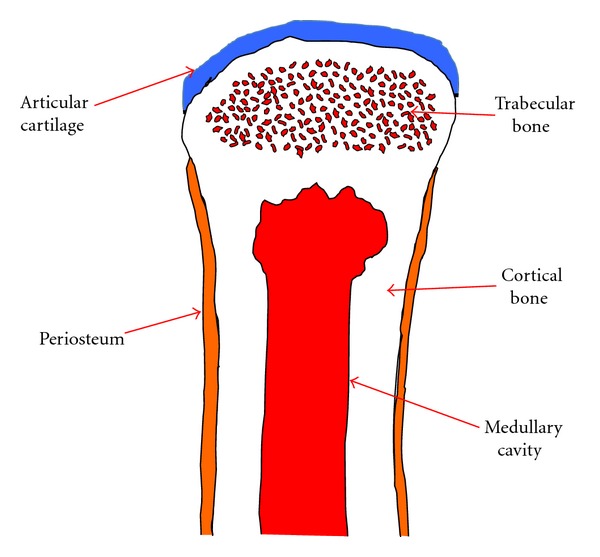
Schematic diagram of a portion of a long bone showing the articular cartilage, trabecular bone, cortical bone, medullary cavity, and periosteum.

We acquired several intact, porcine femurs from the surgical labs at Dartmouth Hitchcock Medical Center from animals that had been euthanized according to IACUC (Institutional Animal Care and Use Committee) approved protocols. The bones were mechanically stripped of all flesh using surgical blades and then mounted in a bench vise under a ventilation hood. Two 2 cm diameter by roughly 3 cm long cylindrical specimens were recovered from both femur ends using a hole saw to extract only trabecular bone samples. The remaining cortical shells at either end were cut off with a simple coping saw. The bone samples were then soaked in formalin for 4 weeks.  Figure 1 shows a schematic diagram of one end of a long bone illustrating the trabecular bone in relation to the other components.

We applied a standard protocol used in the Anatomy Department at Dartmouth Medical School for removing the embedded marrow. This involved soaking each sample in commercially available ammonia (3% solution) baths for two weeks, followed by full-week immersions in 75%, 50%, 25% and 0% ammonia solutions (percentages with respect to the original 3% solution). During each week, the solutions were repeatedly refreshed to maximize the solvent effect. The progressive decrease in ammonia concentrations was designed to dissolve all of the marrow (high concentration of fatty material) even within the deepest portions of the trabecular bone specimens while also minimizing potential damage to the intricate trabecular structure. The first set of micro-CT images at the start of the experiments verified that the marrow had been completely removed.

### 2.2. Bone Testing Procedure

**Figure 2:**
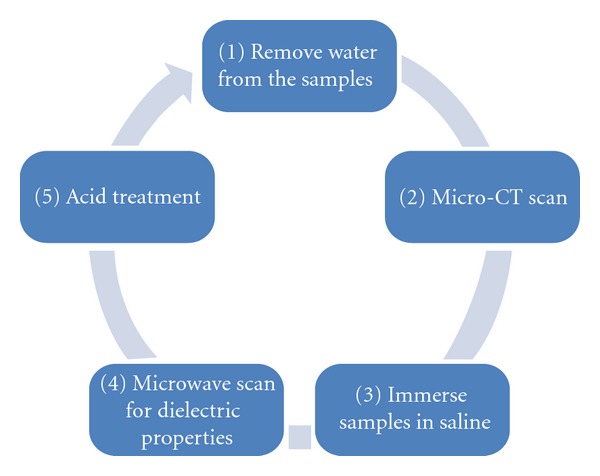
Experimental procedure flow for investigating the *in vitro *relationship between bone mineral and dielectric properties of porcine trabecular bone specimens.

The flow graph in Figure 2 illustrates the procedures used for testing and processing the demarrowed bone samples. These steps are described in more detail below.

**(1) Removal of All Water from the Samples**

This involved placing the samples in a custom desicator attached to an in-house vacuum source for a minimum of 4 days. We also documented the weight, length, and diameter of each sample at this step.

**(2) Micro-CT of Each Bone Sample**

**Figure 3:**
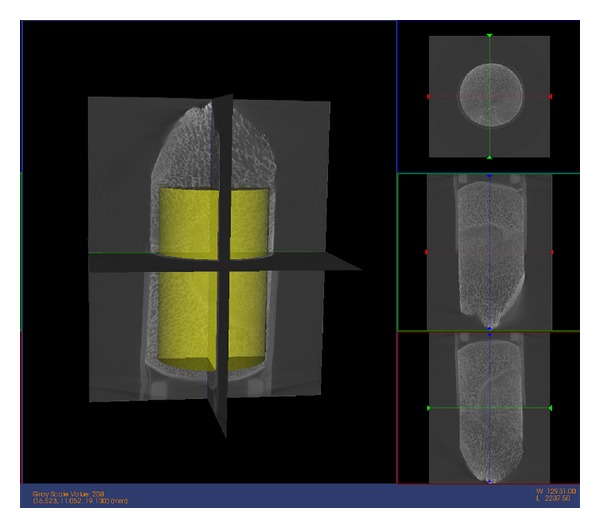
Micro-CT scan of a sample bone specimen. The yellow cylinder is the region of interest for extracting bone mineral parameters. The size of the ROI is kept constant in the longitudinal study for each specimen. The ROI is located at approximately the same position to minimize variation.

We used a GE eXplore Locus SP micro-CT system with GE MicroView 2.1.2 software for reconstructing and analyzing the images. The protocol included using an 80 KeV/80 mAmp X-ray source, and data was acquired with a 0.5° increment between views. Photographs were taken of all samples in the X-ray chamber to note the various physical landmarks (growth plate and uneven height features) for registration with the microwave imaging tests. The baseline resolution for this configuration was 28 microns; however, because we applied a 2 × 2 binning strategy during the reconstruction process to reduce noise, the effective resolution was reduced to 56 microns. In this case, we chose cylindrical regions of interest because of the sample shape (Figure 3). The GE analysis algorithms have the capability of calculating values for: BMD-bone mineral density, BMC-bone mineral content, TMD-tissue mineral density, TMC-tissue mineral content, and bone volume fraction (BVF), where BVF = ((Bone Volume)/(Total Sample Volume)) ∗ 100%. According to the manufacturer, both BMD and BMC use parameters set by the user-selected region of interest (ROI), and the resulting bone values are affected by factors other than bone (e.g., soft tissue, air). Having air included in the ROI has particularly deleterious effects; therefore, the TMC and BVF parameters are more biologically meaningful. These values were computed utilizing calibrations against known phantoms supplied by the manufacturer. The data acquisition time was roughly 2.5 hours for full scans while the associated micro-CT image reconstruction process required 10 minutes for each sample.

**(3) Immersion of Bone Samples in Saline**

For microwave imaging purposes, the samples were submerged in a 0.9% saline solution in an 18 mm diameter plastic test tube with 0.5 mm wall thickness for 24 hours. The dielectric properties of the plastic over the range of 900–1300 MHz were *ε*_*r*_ = 2.8 and *σ* = 0.01 S/m. After this, the test tubes were placed in a pressure chamber with a vacuum applied for 10 minutes. This was followed by 10 minutes in the same chamber with 45 lbs/inch^2^ of applied pressure. This two-step procedure was repeated three times as a way of minimizing any trapped air bubbles within the samples.

**(4) Microwave Measurements**

**Figure 4:**
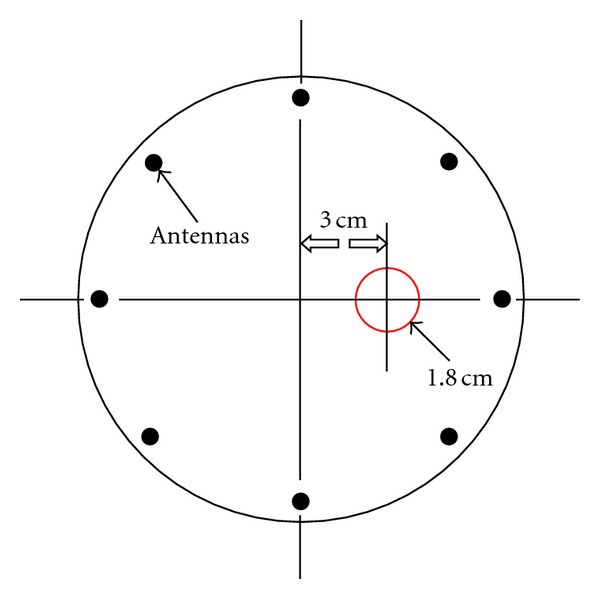
Position of the test tube with respect to the microwave antennas within the imaging tank (top view). (There are 16 antennas in total, only 8 are shown for simplicity. The antennas are positioned equiangularly on a 15.2 cm diameter circle.)

**Figure 5:**
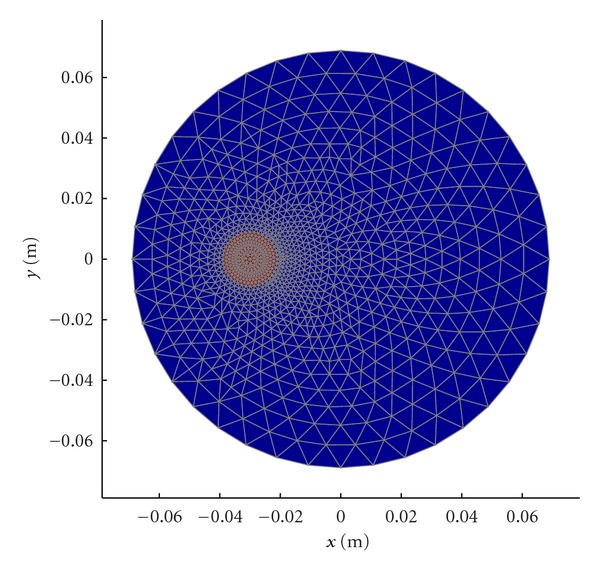
Soft prior mesh for the microwave image reconstruction comprised of 1107 nodes and 2092 triangular elements.

For the dielectric property study, the test tubes were placed at a position 3 cm offset from the center within the illumination chamber (Figure 4). The tank consisted of 16 monopole antennas suspended in an 80 : 20 mixture of glycerin and water which surrounded the bone samples. The dielectric properties of the liquid mixture were *ε*_*r*_ = 28.0, 24.9, 22.5, and *σ* = 1.01, 1.19, 1.35 S/m for 900, 1100, and 1300 MHz, respectively. Each antenna individually broadcasts a single-frequency, continuous wave signal with the remaining 15 antennas acting as receivers. This process was repeated for all 16 antennas acting as the transmitter and at 11 frequencies (500–2500 MHz at a step size of 200 MHz) to produce 2640 measurements (16 transmitters × 15 receivers × 11 frequencies). This data was then used in our Gauss-Newton 2D imaging algorithm while applying our soft prior regularization to extract accurate reconstructions of the properties within the test tube zone. The soft prior reconstruction mesh (Figure 5) illustrates the isolation of the test tube region from the remaining homogeneous coupling bath. (The test tube region was on the left, instead of on the right in Figure 4 because the reconstruction software is oriented for viewing from the bottom of the tank to the top.) It is essentially a mirror image of that observed from the top. This mesh information is then included with the reconstruction algorithm to provide nearly homogeneous values for the target zone (note, the properties of the surrounding zone are known from direct measurements). Single-frequency images were recovered in under 2 minutes while allowing the algorithm to converge for 20 iterations. The bone samples were then rinsed for 10 minutes in tap water before the next process.

We investigated whether orientation of the test tube had any deleterious effect on our measurements. For several test cases, the test tube containing the bone specimen was rotated 0°, 90°, 180°, and 270° and scanned with the microwave tomographic imaging (MTI) system. The standard deviations for relative permittivity and conductivity were 0.35 and 0.026 S/m, respectively. Even with this low level of variation, orientation was taken into consideration during the process. Each bone specimen was marked with ink and maintained in the same direction for all scans.

**(5) Acid Treatment**

The bone samples were suspended in a 5% nitric acid (bone decalcifier) solution for approximately 2 hours to remove a controlled percentage of the mineralization. We determined that most bone samples typically began with roughly 40% mineral density and that each 2.1-hour acid treatment decreased the density in increments of approximately 10%. The protocol of acid treatment allowed us to recover 5 measurements for each sample at different mineral densities. All samples were rinsed in tap water for 10 minutes after each session to terminate the oxidation process.

### 2.3. Microwave Imaging with Soft Prior Regularization

**Figure 6:**
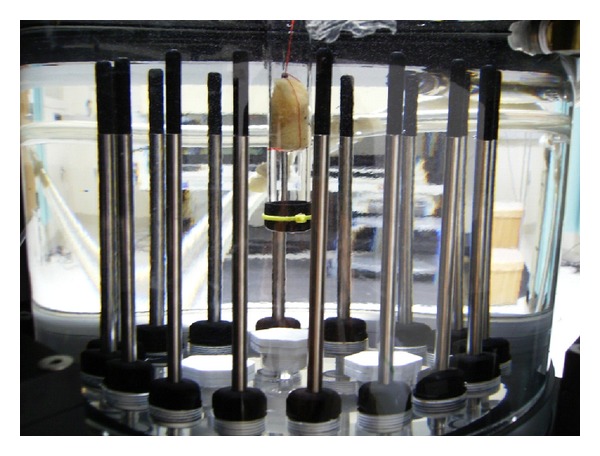
Microwave data acquisition of a saline-saturated bone sample in a test tube submerged in an 80% glycerin bath (side view).

Figure 6 shows the microwave imaging configuration for interrogating the trabecular bone samples. As described above, each sample was immersed in a 0.9% saline solution within an 18 mm diameter test tube. The reconstruction mesh (Figure 5) only encompasses the sample domain since the properties of the surrounding liquid are known and are not reconstructed. The minimization statement for the Gauss-Newton reconstruction utilizing the soft prior regularization approach is given as

(1) $$\min{||\Gamma^{m}-\Gamma^{c}\left(k^{2}\right)||}^{2}+{||\Phi^{m}-\Phi^{c}\left(k^{2}\right)||}^{2}+\lambda L\left\{k^{2}-k_{\text{bk}}^{2}\right\},$$

where Γ^*m*^ and Γ^*c*^ are the measured and computed log magnitude values, Φ^*m*^ and Φ^*c*^ are the measured and computed phase values, *k* ^2^ is the complex wave number squared distribution at the current iteration, *k*_bk_^2^ is the wave number squared for the surrounding background coupling medium, *λ* is the soft prior regularization parameter and *L* is the soft prior matrix. *k* ^2^ comprises the permittivity and conductivity images through the relationship *k* ^2^ = *ω* ^2^ *με* + *jωμσ*, where *ω* is the frequency in radians, *μ* is the magnetic permeability, and *ε* and *σ* are the electrical permittivity and conductivity, respectively. Note that the first two terms are essentially the implementation of our log transformation in the Gauss-Newton algorithm which is described in detail in Meaney et al. [37]. (All of the details of this approach are described in more detail in Golnabi et al. [35].) The critical aspect of the soft prior regularization is the composition of the matrix *L*

(2) $$\begin{array}{c} R_{1}\{\left[\begin{array}{ccccccccc} 1 & -\frac{1}{N_{1}} & \cdots & -\frac{1}{N_{1}} & 0 & \cdots & \cdots & \cdots & 0\\ -1 & 1 & \cdots & -\frac{1}{N_{1}} & \cdots & \cdots & \cdots & \cdots & \cdots \\ \cdots & \cdots & \cdots & \cdots & \cdots & \cdots & \cdots & \cdots & \cdots \\ -\frac{1}{N_{1}} & -\frac{1}{N_{1}} & \cdots & 1 & 0 & \cdots & \cdots & \cdots & 0 \end{array}\right]\\ R_{2}\{\left[\begin{array}{ccccccccc} 0 & \cdots & \cdots & 0 & 1 & -\frac{1}{N_{2}} & \cdots & \cdots & -\frac{1}{N_{2}}\\ \cdots & \cdots & \cdots & \cdots & -\frac{1}{N_{2}} & 1 & \cdots & \cdots & \cdots \\ \cdots & \cdots & \cdots & \cdots & \cdots & -\frac{1}{N_{2}} & \cdots & \cdots & \cdots \\ \cdots & \cdots & \cdots & \cdots & \cdots & \cdots & \cdots & \cdots & \cdots \\ \cdots & \cdots & \cdots & \cdots & \cdots & \cdots & \cdots & 1 & -\frac{1}{N_{2}}\\ 0 & \cdots & \cdots & 0 & -\frac{1}{N_{2}} & \cdots & \cdots & -\frac{1}{N_{2}} & 1 \end{array}\right] \end{array}$$

Equation (2) shows the *L* matrix constructed with regions *R*_1_ and *R*_2_ grouped accordingly. As shown in Figure 5, *R*_1_ is the background region (blue), and *R*_2_ is the region that has the bone specimen (red). There are *N*_1_ and *N*_2_ nodes in regions *R*_1_ and *R*_2_, respectively. Based on the formation of the regularization matrix *L*, *L* ^*T*^ *L* can be viewed as an approximation of a second-order Laplacian smoothing operator inside each region that limits the smoothing across the boundary of distinct regions [38].

## 3. Results

**Figure 7:**
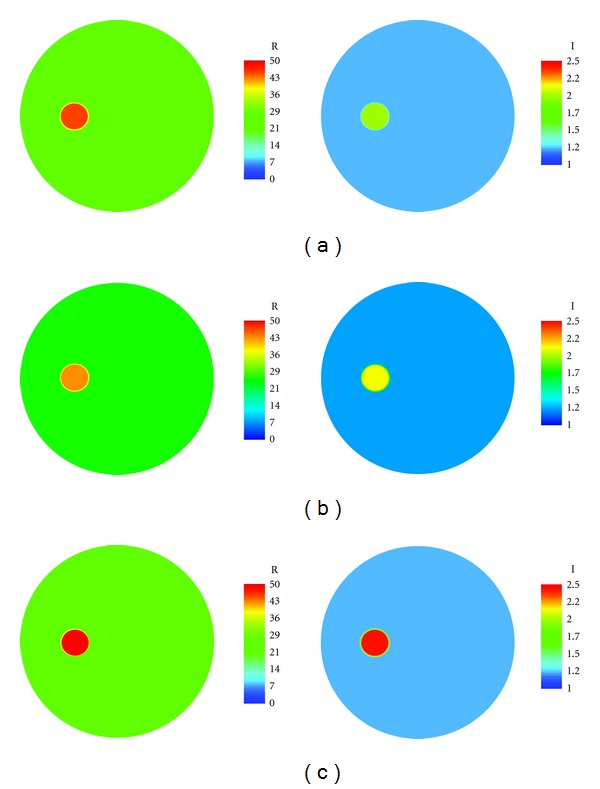
Reconstructed permittivity images (left) of the (a) 1st, (b) 2nd, and (c) 5th microwave scan of a saline-saturated bone specimen in a test tube at 1100 MHz, respectively. The green background shows the permittivity of the coupling liquid (80% glycerin) inside the imaging tank. The images on the right are the corresponding conductivity images.

We used six bone specimens in this experiment, and each was scanned by both the micro-CT and microwave tomography systems between intervening demineralization treatments. For all samples, the dry mass and diameters decreased 50.90% and 12.17% on average, respectively, with associated standard deviations of 5.88% and 2.72%, respectively.  Figure 7 shows representative 1100 MHz soft prior permittivity and conductivity images at the (a) first, (b) second, and (c) final imaging scans. The bath was the same for all sessions as illustrated by the backgrounds of each image being identical to the others. It is interesting to note that while the dry diameters shrunk slightly from the acid treatments, when the samples were immersed in the saline, they expanded to fit snuggly against the test tube walls. The properties of the recovered targets demonstrated a clear increasing trend from the first to the last imaging session.

**Figure 8:**
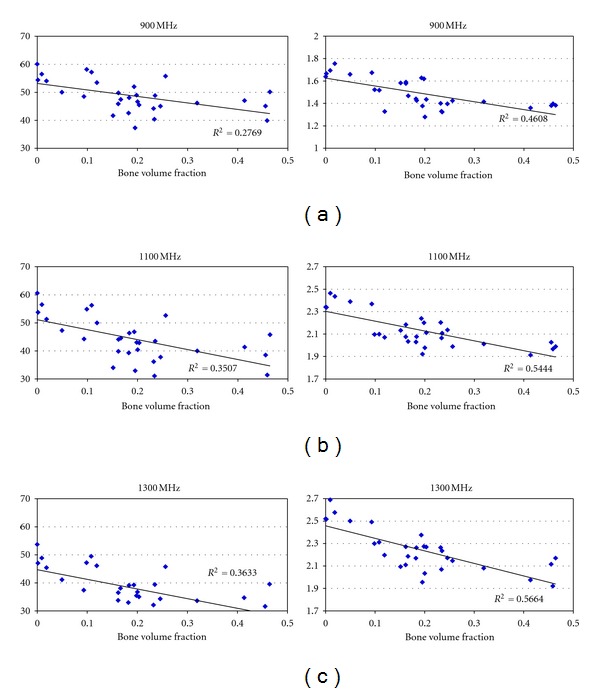
Scatter plot of dielectric properties versus bone volume fraction for saline-saturated bone specimens for different frequencies (*n* = 30).

The data for the six samples were pooled and analyzed.  Figure 8 shows the scatter plots for the dielectric properties versus BVF at three frequencies (0.9, 1.1, and 1.3 GHz). Overall, both properties show a distinct downward trend with respect to bone volume fraction for all frequencies. The *P* values for both relative permittivity and conductivity at all three frequencies are less than the 0.05 threshold for significance. These relationships are further confirmed with the Pearson product-moment correlation coefficients (PMCC) summarized in Table 1. (The Pearson coefficients are a measure of the strength of linear dependence between two variables and are defined as the covariance of the two variables divided by the product of their standard deviations.) The correlation coefficients for the relative permittivity and conductivity with respect to BVF are above the threshold (in terms of their absolute values) for statistical significance with *n* = 30. The conductivity Pearson coefficients are consistently higher than those for the permittivity suggesting a higher level of correlation.

## 4. Discussion and Conclusions 

We have successfully applied our microwave imaging system to assess the dynamic dielectric properties of bone tissue samples. This is particularly important in the setting of measuring bone samples where other techniques such as open-ended dielectric probes have failed. The results are consistent with what would be predicted by Maxwell-Fricke mixture laws [4]; that is, with the decreased mineralization (low dielectric property values) and increased saline (higher properties), the bulk dielectric properties of the saline-saturated bone sample increased. They are consistent with the findings from Chakkalakal et al. in that the variations in dielectric properties are determined by the content in the pore holes [21], although the operating frequencies and variation mechanisms of dielectric property dispersions differ. The results further confirm the flexibility and strength of the soft prior regularization strategy in the context of microwave imaging. 

In addition, these results suggest a mechanism to explain the correlation between bone dielectric properties and age observed by Peyman et al. [11]. While these relationships were hypothesized because of the dynamic nature of bone tissue and the heavy dependence of tissue dielectric properties on water content [3], it was essential that these results be confirmed in direct investigations. 

The measurement conditions for these experiments were suboptimal with respect to assessing the natural property variations as functions of bone density. In the body, the pore holes are filled with blood vessels and marrow. A future study should extend these measurements to *in vivo* or freshly excised bones to determine the contribution of these materials in the pore holes to the dielectric properties. The bone density variations could be induced directly by varying the animal diets, especially the mineral content, to provide more realistic controls. Notwithstanding, these results are a promising first step towards understanding the relationship between dielectric properties and bone density metrics and set the stage for exploiting this mechanism for microwave bone imaging as a clinical diagnostic tool. 

## Figures and Tables

**Table 1:** Values for Pearson product-moment correlation coefficient of the dielectric properties and BVF.

Frequency (GHz)	Pearson *r*	Pearson *r*	Threshold for statistical significance (*n* = 30)
	*ε*_*r*_ versus BVF	*σ* versus BVF	
0.9	−0.53	−0.68	−0.37
1.1	−0.59	−0.74	−0.37
1.3	−0.60	−0.75	−0.37
